# Lipid Effects of Icosapent Ethyl in Women with Diabetes Mellitus and Persistent High Triglycerides on Statin Treatment: ANCHOR Trial Subanalysis

**DOI:** 10.1089/jwh.2017.6757

**Published:** 2018-09-01

**Authors:** Eliot A. Brinton, Christie M. Ballantyne, John R. Guyton, Sephy Philip, Ralph T. Doyle, Rebecca A. Juliano, Lori Mosca

**Affiliations:** ^1^ Utah Lipid Center, Salt Lake City, Utah.; ^2^ Department of Medicine, Baylor College of Medicine and the Houston Methodist DeBakey Heart and Vascular Center, Houston, Texas.; ^3^ Department of Endocrinology, Metabolism, and Nutrition, Duke University School of Medicine, Durham, North Carolina.; ^4^ Medical Affairs, Amarin Pharma, Inc., Bedminster, New Jersey.; ^5^ Clinical Development, Amarin Pharma, Inc., Bedminster, New Jersey.; ^6^ Department of Medicine, Columbia University Medical Center, New York, New York.

**Keywords:** diabetes mellitus, hypertriglyceridemia, cardiovascular diseases, eicosapentaenoic acid, women's health

## Abstract

***Background:*** High triglycerides (TG) and diabetes mellitus type 2 (DM2) are stronger predictors of cardiovascular disease (CVD) in women than in men, but few randomized, controlled clinical trials have investigated lipid-lowering interventions in women and none have reported results specifically in women with high TG and DM2. Icosapent ethyl (Vascepa) is pure prescription eicosapentaenoic acid (EPA) ethyl ester approved at 4 g/day as an adjunct to diet to reduce TG ≥500 mg/dL.

***Methods:*** The 12-week ANCHOR trial randomized 702 statin-treated patients (73% with DM; 39% women) at increased CVD risk with TG 200–499 mg/dL despite controlled low-density lipoprotein cholesterol (LDL-C; 40–99 mg/dL) to receive icosapent ethyl 2 g/day, 4 g/day, or placebo. This *post hoc* analysis included 146 women with DM2 (97% white, mean age 62 years) randomized to icosapent ethyl 4 g/day (*n* = 74) or placebo (*n* = 72).

***Results:*** Icosapent ethyl significantly reduced TG (−21.5%; *p* < 0.0001) without increasing LDL-C and lowered other potentially atherogenic lipid/lipoprotein, apolipoprotein, and inflammatory parameters versus placebo. Icosapent ethyl increased EPA levels in plasma (+639%; *p* < 0.0001; *n* = 49) and red blood cells (+599%; *p* < 0.0001; *n* = 47) versus placebo. Safety and tolerability of icosapent ethyl were generally similar to placebo.

***Conclusion:*** In women with DM2 at high CVD risk with persistently high TG on statins, icosapent ethyl 4 g/day reduced potentially atherogenic parameters with safety and tolerability comparable to placebo. Potential CVD benefits of icosapent ethyl are being tested in ∼8000 men and women at high CVD risk with high TG on statins in the ongoing Reduction of Cardiovascular Events with Icosapent Ethyl - Intervention Trial (REDUCE-IT) cardiovascular (CV) outcome trial.

## Introduction

Cardiovascular disease (CVD) has long been the leading cause of morbidity and mortality in women (as in men), higher than all forms of cancer combined.^1^ Fortunately, there is now growing public awareness of the preeminence of CVD in women,^1–4^ and CVD-related death rates have decreased by nearly one-half among US adults over the past few decades. Unfortunately, there has been less decline in women than in men, and these rates remain higher in women than men in some race/ethnic groups.^1,4,5^ Furthermore, the overall downward trend in CVD-related deaths has plateaued in recent years, and in 2014 and 2015 modest upticks in both men and women were reported.^1,6^

Importantly, diabetes mellitus type 2 (DM2), a key contributory factor in CVD risk, is increasing in both women and men in the United States and many other countries.^1,7^ DM2 confers a greater relative increase in risk of CVD in women compared with men, and a large meta-analysis indicated a 44% higher relative risk ratio for coronary heart disease due to DM2 in women compared with men.^8–10^ Furthermore, women with DM2 have been shown to have a higher adjusted hazard ratio (HR) of fatal coronary artery disease (HR = 14.7; 95% CI, 6.2–35.3) compared with men with DM2 (HR = 3.8; 95% CI, 2.5–5.7),^11^ and in patients with DM2, CVD rates have declined less in women than in men.^12^ Thus, addressing the excess CVD risk in women with DM2 is a crucial component in the overall prevention and management of CVD risk.^9^

Likewise, elevated triglycerides (TG) are usually found to have a greater relative adverse effect on CVD in women than in men,^13,14^ although relatively little is known about the contribution of high TG to the excess CVD risk found in women with DM2. A major causal factor of high TG is DM2, especially when glycemia is poorly controlled^15^; conversely, high TG are a major risk factor for insulin resistance and, thus, in the development of new-onset DM2.^16^ Despite this interaction, and the importance of each factor in CVD risk in women, there is a lack of data documenting safe and effective strategies for reducing TG in women with DM2.

Together, these findings underscore the need to identify effective strategies to reduce CVD risk in women having both DM2 and elevated TG. Some of the challenges in CVD risk management in women were highlighted in recent surveys by the Women's Heart Alliance, which found that women's awareness of CVD risk is low (45% being unaware that CVD is the number 1 killer of women) and, surprisingly, that CVD prevention in women is not a top concern for most physicians.^17^ Furthermore, only 22% of primary care physicians felt well prepared to assess, much less manage, women's CVD risk.^17^

Another ongoing challenge in addressing increased CVD risk in women is the lack of published data regarding the efficacy and safety of treatments for CVD prevention in women, since controlled clinical trials have historically under-enrolled, or even excluded, women.^4,18^ Known differences in pathophysiology and clinical manifestations of CVD in women versus men strongly suggest that a given clinical treatment cannot be assumed to yield equal efficacy or safety for women as for men.^4,18^ Recently, as awareness of the importance of CVD in women has grown, enrollment of representative numbers of female patients in lipid and cardiovascular trials has achieved higher priority in trial design.^4,18^

Icosapent ethyl (a pure ethyl ester of eicosapentaenoic acid [EPA]; Vascepa^®^, Amarin Pharma, Inc., Bedminster, NJ) is a prescription omega-3 fatty acid therapy approved by the Food and Drug Administration (FDA) at a dose of 4 g/day as an adjunct to diet to reduce TG in adults with severe hypertriglyceridemia (TG ≥500 mg/dL).^19^ In the ANCHOR trial, icosapent ethyl was safe and effective in reducing TG and other atherogenic parameters in adults with high TG (200–499 mg/dL) on stable statin therapy.^20^ These effects were also seen in the subgroup of patients with DM2^21^ and separately in the subgroup of all female subjects.^20,22^ The purpose of this analysis was to examine effects of icosapent ethyl on TG and other atherogenic factors in women with DM2 who participated in the ANCHOR trial.

## Methods

### Trial design and participants

Details regarding trial design, including participant criteria, of the ANCHOR trial were previously reported.^20^ In brief, ANCHOR was a phase 3, multicenter, placebo-controlled, randomized, double-blind, 12-week clinical trial conducted in the United States from December 2009 through February 2011. Patients with high CVD risk and high TG (200–499 mg/dL) despite controlled low-density lipoprotein cholesterol (LDL-C; ≥40 and <100 mg/dL) on stable statin therapy (with or without ezetimibe) were included in the trial. High CVD risk was defined as a history of coronary artery disease (*i.e*., history of myocardial infarction, unstable or stable angina, coronary artery procedures, or clinically significant myocardial ischemia), noncoronary forms of clinical atherosclerosis, or DM1 or DM2. Patients were randomized to icosapent ethyl 4 g/day, 2 g/day, or placebo. This current *post hoc* subgroup analysis includes all women from ANCHOR who had DM2 and who received the FDA-approved dose of 4 g/day or placebo.

### Efficacy assessments

The primary efficacy variable was the median difference in percent change in plasma TG from baseline to week 12 between icosapent ethyl 4 g/day and placebo. Additional assessments included the median difference in percent change from baseline to week 12 between icosapent ethyl 4 g/day and placebo in plasma levels of LDL-C, non-high-density lipoprotein cholesterol (non-HDL-C), total cholesterol (TC), HDL-C, very-LDL-C (VLDL-C), VLDL-TG, remnant lipoprotein cholesterol (RLP-C), apolipoprotein B (Apo B), apolipoprotein C-III (Apo C-III), oxidized LDL (ox-LDL), lipoprotein-associated phospholipase A_2_ (Lp-PLA_2_), and high-sensitivity C-reactive protein (hsCRP), as well as plasma and red blood cell (RBC) concentrations of EPA. These parameters were measured as previously described.^21,23–27^ In ANCHOR, these EPA levels were measured in approximately the first 216 patients with complete sample sets.

### Safety assessments

Safety assessments included treatment-emergent adverse events (TEAEs), which were defined as any adverse events (AEs) that began after the first dose of trial medication or that occurred before the first dose and worsened in severity during the double-blind treatment period. TEAEs reported in this analysis include total TEAEs and those occurring in >3% in any group in the full population of the ANCHOR trial across treatment arms (nausea, diarrhea, nasopharyngitis, and arthralgia).^20^

### Statistical analyses

The ANCHOR protocol included prespecified subgroup analyses of the primary efficacy variable; randomization of patients was stratified by gender, statin type (atorvastatin, rosuvastatin, or simvastatin), and the presence or absence of DM (DM1 or DM2) at baseline. The current subgroup analysis of women with DM2 was not prespecified. Both the prespecified and *post hoc* efficacy analyses were primarily done in a modified intent-to-treat population, defined as all randomized patients who had a baseline efficacy measurement, received ≥1 dose of trial drug, and had ≥1 postrandomization efficacy measurement. Median difference in percent change from baseline between icosapent ethyl 4 g/day and placebo for the primary efficacy variable and additional assessments was estimated with the Hodges–Lehmann method (*p* values from the Wilcoxon rank-sum test for treatment comparisons) where departures from normal distribution were observed; for normally distributed parameters, an analysis of covariance model was used with least squares (LS) mean and standard error (SE). ANCHOR was designed to have greater than 90% power to detect a difference of 15% between icosapent ethyl 4 g/day and placebo in percent change from baseline in fasting TG and 80% power to demonstrate noninferiority of LDL-C response between icosapent ethyl 4 g/day and placebo, within a 6% margin. For all prespecified subgroup analyses and all *post hoc* analyses (including those reported in this study), 0.05 was the prespecified alpha for significance.

## Results

### Patients

Overall, the ANCHOR trial randomized 702 statin-treated patients to icosapent ethyl 4 g/day, 2 g/day, or placebo. Of the total population, 39% were women^20^ and 32% were women with DM2 (none of the women had DM1). The current *post hoc* subgroup analysis includes the 146 women with DM2 randomized to receive icosapent ethyl 4 g/day (*n* = 74) or placebo (*n* = 72). Baseline characteristics of women with DM2 from the ANCHOR trial are shown in Table 1. Among these, eight in the icosapent ethyl 4 g/day group discontinued treatment (four due to AEs, two withdrew consent, one lost to follow-up, and one for another reason) and seven discontinued in the placebo group (three due to AEs, three withdrew consent, and one for another reason).

**Table 1.** Baseline Demographics of Women with Diabetes Mellitus Type 2 from the ANCHOR Trial

*Variable*	*Icosapent ethyl 4 g/day (*n* = 74)*	*Placebo (*n* = 72)*
Age, mean (SD), years	61.2 (9.4)	62.1 (9.7)
Body mass index, mean (SD), kg/m^2^	32.6 (4.9)	33.2 (5.3)
White, *n* (%)	73 (98.7)	69 (95.8)

SD, standard deviation.

### Efficacy

**FIG. 1.**
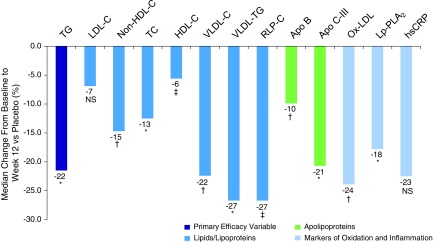
Percent change in atherosclerosis risk parameters with icosapent ethyl 4 g/day in women with DM2 from the ANCHOR trial. Values represent median difference in percent change from baseline for icosapent ethyl 4 g/day versus placebo. *p < 0.0001; ^†^*p* < 0.001; ^‡^*p* < 0.05; NS, not significant versus placebo. Apo C-III, apolipoprotein C-III; Apo B, apolipoprotein B; DM, diabetes mellitus; non-HDL-C, non-high-density lipoprotein cholesterol; hsCRP, high-sensitivity C-reactive protein; LDL-C, low-density lipoprotein cholesterol; ox-LDL, oxidized low-density lipoprotein; RLP-C, remnant lipoprotein cholesterol; TC, total cholesterol; TG, triglycerides; VLDL-C, very-low-density lipoprotein cholesterol; VLDL-TG, very-low-density lipoprotein triglycerides.

There was a significant reduction of 21.5% (*p* < 0.0001) in fasting TG (primary endpoint) in women with DM2 treated with icosapent ethyl 4 g/day compared with placebo (Table 2 and Fig. 1). This reduction was similar to that observed in men with DM2 (24.4%; *p* < 0.0001 vs. placebo). Regarding changes in other lipid levels compared with placebo, significant reductions in non-HDL-C, TC, VLDL-C, VLDL-TG, RLP-C, and HDL-C were also observed, as noted in Table 2 and Figure 1. Apo B and Apo C-III were also significantly reduced compared with placebo, although the trend to a decrease in LDL-C did not reach statistical significance (Table 2 and Fig. 1). Regarding changes in markers of oxidation and inflammation, ox-LDL and Lp-PLA_2_ were significantly reduced compared with placebo, and while there was a trend toward decreased hsCRP, it did not reach statistical significance (Table 2 and Fig. 1). Overall, the reductions in the efficacy parameters assessed in women with DM2 were similar to those in men with DM2, with the exception of ox-LDL (data not shown).

**Table 2.** Effects of Icosapent Ethyl 4 g/day on Atherosclerosis Risk Factors in Women with Diabetes Mellitus Type 2 from the ANCHOR Trial

	*Icosapent ethyl 4 g/day*			*Placebo*			
*Parameter*	*Baseline*	*End of treatment*	*Change from baseline, %*	*Baseline*	*End of treatment*	*Change from baseline, %*	*Median difference in % change from baseline for icosapent ethyl 4 g/day* versus *placebo, %*
Lipid/lipoprotein parameters							
TG (mg/dL) (primary efficacy variable)							
*n* = 70, 70	259 (94.5)	229 (78.5)	−17.4 (33.6)	260 (79.5)	286 (123)	5.0 (40.5)	−21.5 <0.0001
LDL-C (mg/dL)							
*n* = 70, 70	85.5 (24.0)	88.5 (26.0)	0.6 (26.2)	81.5 (25.0)	84.5 (35.0)	11.4 (30.6)	−6.9 0.1298
Non-HDL-C (mg/dL)							
*n* = 70, 70	132 (30.0)	124 (35.0)	−5.5 (19.1)	126 (37.0)	135 (40.0)	11.2 (27.1)	−14.7 0.0002
TC (mg/dL)							
*n* = 70, 70	178 (33.0)	170 (33.0)	−5.2 (16.2)	170 (38.0)	184 (43.0)	8.6 (17.5)	−12.5 <0.0001
HDL-C (mg/dL)							
*n* = 70, 70	41.0 (13.0)	40.0 (13.0)	−2.5 (21.9)	41.5 (15.0)	44.0 (18.0)	6.2 (23.0)	−5.6 0.0344
VLDL-C (mg/dL)							
*n* = 70, 70	41.0 (20.0)	37.0 (23.0)	−18.6 (44.8)	42.0 (21.0)	46.5 (32.0)	6.4 (55.7)	−22.4 0.0005
VLDL-TG (mg/dL)							
*n* = 70, 70	183 (78.0)	146 (78.0)	−22.7 (29.4)	192 (98.0)	201 (127)	4.6 (68.1)	−26.7 <0.0001
RLP-C (mg/dL)^a^							
*n* = 21, 25	13.0 (5.0)	11.0 (6.0)	−27.3 (54.4)	15.0 (8.0)	14.0 (11.0)	13.0 (76.7)	−26.7 0.0315
Apolipoprotein parameters							
Apo B (mg/dL)^b^							
*n* = 65, 65	97.0 (19.0)	91.0 (26.0)	−2.2 (15.0)	92.0 (23.0)	98.0 (33.0)	7.1 (22.4)	−9.9 0.0008
Apo C-III (mg/dL)^b^							
*n* = 65, 61	15.5 (4.1)	14.3 (4.0)	−10.4 (26.3)	15.2 (3.9)	16.5 (4.6)	10.3 (28.4)	−20.7 <0.0001
Markers of oxidation and inflammation							
Ox-LDL (U/L)^a^							
*n* = 25, 26	55.6 (10.4)	52.9 (10.3)	−9.6 (25.3)	56.3 (16.5)	66.1 (20.4)	19.1 (17.7)	−23.9 0.0002
Lp-PLA_2_ (ng/mL)							
*n* = 65, 63	177 (49.0)	156 (41.0)	−14.5 (19.0)	182 (64.0)	193 (52.0)	3.9 (22.1)	−17.8 <0.0001
hsCRP (mg/L)							
*n* = 65, 65	2.9 (2.8)	2.6 (3.8)	7.4 (82.5)	3.7 (4.1)	3.8 (5.6)	21.4 (96.5)	−22.5 0.0559

Data are presented as median (interquartile range) for endpoint values. Median percent changes versus placebo are Hodges–Lehmann medians. *p* Values are from Wilcoxon rank-sum test. Patient numbers are presented as icosapent ethyl 4 g/day and placebo, respectively.

^a^ RLP-C and ox-LDL were only measured in approximately the first 35% of patients randomized in ANCHOR.

^b^ Apo B and Apo C-III levels were measured in the subset of all patients with available archived plasma samples from ANCHOR.

Apo C-III, apolipoprotein C-III; Apo B, apolipoprotein B; non-HDL-C, non-high-density lipoprotein cholesterol; hsCRP, high-sensitivity C-reactive protein; LDL-C, low-density lipoprotein cholesterol; Lp-PLA_2_, lipoprotein-associated phospholipase A_2_; ox-LDL, oxidized low-density lipoprotein; RLP-C, remnant lipoprotein cholesterol; TC, total cholesterol; TG, triglycerides; VLDL-C, very-low-density lipoprotein cholesterol; VLDL-TG, very-low-density lipoprotein triglycerides.

### Plasma and RBC EPA levels

EPA content in plasma and RBC was measured in a subset of women in the icosapent ethyl 4 g/day group (*n* = 23 and *n* = 22, respectively) and placebo group (*n* = 26 and *n* = 25, respectively). Icosapent ethyl 4 g/day significantly increased mean (standard deviation [SD]) plasma EPA levels from a baseline value of 24.4 (8.1) to 182.4 (76.4) μg/mL at 12 weeks, an LS mean (SE) increase of 638.5% (68.1%) versus placebo (*p* < 0.0001). Icosapent ethyl 4 g/day also significantly increased mean (SD) EPA levels in RBCs from a baseline value of 10.7 (5.5) to 65.7 (32.9) μg/mL, an LS mean (SE) increase of 598.5% (75.5%) versus placebo (*p* < 0.0001).

### Adverse events

TEAEs were reported in 36 (48.6%) women with DM2 in the icosapent ethyl 4 g/day group and 36 (50.0%) in the placebo group. In the subgroup of women with DM2 from ANCHOR (icosapent ethyl 4 g/day vs. placebo groups, respectively), two (2.7%) versus two (2.8%) reported nausea; four (5.4%) versus seven (9.7%) reported diarrhea; one (1.4%) versus two (2.8%) reported nasopharyngitis; and two (2.7%) versus none experienced arthralgia. Four women with DM2 discontinued trial treatment due to AEs in the icosapent ethyl 4 g/day group, one each due to loose stools, subarachnoid hemorrhage, gastroesophageal reflux, and lip swelling (the gastroesophageal reflux and loose stools being considered related to trial medication), while three discontinued for AEs in the placebo group, one each due to abdominal pain, headache, and facial rash (the facial rash being considered related to trial medication).

## Discussion

Icosapent ethyl 4 g/day reduced TG without increasing LDL-C and reduced other potentially atherogenic lipid/lipoprotein, apolipoprotein, and inflammatory parameters versus placebo in the subgroup of women from the ANCHOR trial with DM2 and persistently elevated TG despite statin therapy. These reductions were generally similar to those of the overall ANCHOR population,^20,24,27,28^ all women from ANCHOR,^22^ the subgroup of all patients with DM2,^21^ and the subgroup of men with DM2 (data not shown).

### EPA-only versus EPA plus docosahexaenoic acid therapy

Important points for consideration in the treatment of elevated TG include differences between pure EPA-only therapy versus other prescription omega-3 agents, which contain docosahexaenoic acid (DHA) in addition to EPA. Pure EPA does not raise LDL-C in subjects with high TG^20^ or very high TG.^23^ This finding has been substantiated in women with DM2 in the current analysis. The other prescription agents differ in their lipid effects from pure EPA in two ways. First, these agents may increase LDL-C in patients with elevated TG^29–31^ and may even do so in patients with normal TG,^32^ which could interfere with achievement of LDL-C treatment goals. Second, prescription EPA plus DHA products tend to increase HDL-C,^29–32^ whereas the current analysis showed a modest but statistically significant decrease in HDL-C with pure EPA (Table 2 and Fig. 1). This decrease is comparable to that seen in the entire ANCHOR population^20^ and in the MARINE trial of icosapent ethyl in patients with very high TG at baseline.^23^ The decrease in HDL-C with DHA-free icosapent ethyl could be considered adverse, but might not be so in light of reports that the addition of EPA to reconstituted HDL *in vitro*^33^ and icosapent ethyl treatment *in vivo*^34^ may both enhance antioxidant and anti-inflammatory HDL function. Further research is needed to explore the net clinical effects, if any, of the above changes in LDL-C and HDL-C concentration, particles, and function with EPA-only therapy.

### Icosapent ethyl safety

Drug safety in general, and in women in particular, has been a major focus of the FDA and other federal agencies. The overall safety and tolerability profile of icosapent ethyl is well characterized and has been found to be similar to placebo.^20,23^ The only AE reported in >2% of patients and at a rate greater than placebo is arthralgia, which occurred in 2.3% of patients receiving icosapent ethyl versus 1.0% of patients receiving placebo in a pooled analysis of double-blind randomized clinical trials.^19^ Safety data in all women in the ANCHOR trial^22^ and in women with DM2 in the current analysis support the safety of icosapent ethyl in women and are consistent with the safety data observed in men in the ANCHOR trial. In the population of all women and men with DM2 in ANCHOR, there were no significant increases in fasting plasma glucose, hemoglobin A1C, insulin, or homeostasis model assessment–estimated insulin resistance (HOMA-IR) following treatment with icosapent ethyl 4 g/day.^21^

### Clinical relevance

The findings reported herein suggest that icosapent ethyl 4 g/day is a potentially beneficial treatment for CVD risk reduction in women with DM2. This potential risk reduction is being tested formally and directly in the large ongoing Reduction of Cardiovascular Events with Icosapent Ethyl - Intervention Trial (REDUCE-IT) cardiovascular (CV) outcome trial, which is examining CV outcomes in ∼8000 statin-treated men and women at high CVD risk, randomized to receive double-blind treatment with icosapent ethyl 4 g/day versus placebo.^35^ A reduction in CVD events with a lower dose of EPA ethyl esters (1.8 g/day) has already been reported in a Japanese-only population in the JELIS trial.^36^ A notable subanalysis of that trial compared the effects of EPA ethyl esters in patients with impaired glucose metabolism (DM2 or a fasting plasma glucose of 110 mg/dL or higher) versus its effects in normoglycemic patients. In the former patient group, EPA-only treatment resulted in a 22% relative reduction in major coronary events versus the control group (*p* = 0.048), comparable to an 18% relative reduction in normoglycemic patients versus control (*p* = 0.062).^37^ In addition, the reduction in risk with EPA ethyl esters in the overall JELIS population was comparable in women versus men (*p* = 0.43 for interaction).^36^ To our knowledge, subgroup analyses of CVD effects of EPA ethyl esters in women with DM2, the population in the current analysis, have not been done in the JELIS population.

The CVD-related implications of the lipid-related findings of the present analysis will be clarified by the findings of the REDUCE-IT trial, in which a much larger number of women with DM2 (as well as men and patients without DM2) are being tested for the effects of icosapent ethyl 4 g/day on CVD outcomes, with results expected in 2018.^35^

## Limitations

The strength of the findings from this analysis in women with DM2 and high TG on statin therapy is somewhat limited due to the modest sample size and the *post hoc* nature of the analysis. Furthermore, the ANCHOR trial was not designed to determine effects on CVD events.

## Summary and Conclusions

Women with high TG and DM2 are at particularly high CVD risk. This new *post hoc* analysis of women with high TG and DM2 from the ANCHOR trial shows improvement in key CVD risk factors with icosapent ethyl, a pure DHA-free prescription omega-3 drug. It also shows the safety and tolerability of EPA-only treatment, comparable to that in the overall ANCHOR population and other subgroups thereof. The potential for CVD benefits with icosapent ethyl treatment in women and men with or without DM2 but all with high TG and high CV risk is being tested in the ongoing REDUCE-IT CV outcome trial.
